# The Functionality of UDP-Glucuronosyltransferase Genetic Variants and their Association with Drug Responses and Human Diseases

**DOI:** 10.3390/jpm11060554

**Published:** 2021-06-14

**Authors:** Yazun Jarrar, Su-Jun Lee

**Affiliations:** 1 Department of Pharmacy, College of Pharmacy, Alzaytoonah University of Jordan, Amman 11733, Jordan; yazun.jarrar@zuj.edu.jo; 2 Department of Pharmacology and Pharmacogenomics Research Center, College of Medicine, Inje University, Busan 50834, Korea

**Keywords:** metabolism, drug toxicity, genetic variants, UGTs

## Abstract

UDP-glucuronosyltransferases (UGTs) are phase II drug-metabolizing enzymes that metabolize endogenous fatty acids such as arachidonic acid metabolites, as well as many prescription drugs, such as opioids, antiepileptics, and antiviral drugs. The *UGT1A* and *2B* genes are highly polymorphic, and their genetic variants may affect the pharmacokinetics and hence the responses of many drugs and fatty acids. This study collected data and updated the current view of the molecular functionality of genetic variants on *UGT* genes that impact drug responses and the susceptibility to human diseases. The functional information of *UGT* genetic variants with clinical associations are essential to understand the inter-individual variation in drug responses and susceptibility to toxicity.

## 1. Introduction

The UDP-glucuronosyltransferase (UGT) enzymes are phase II drug-metabolizing enzymes that catalyze the glucuronidation reaction. This chemical reaction involves the formation of a covalent bond between the endogenous polar glucuronic acid with drugs and endogenous lipophilic compounds [1]. The glucuronidated compounds have chemical functional groups that accept glucuronic acid. These functional groups include hydroxyl, carboxylic acid, amine, and thiol [2]. The UGTs glucuronidate endogenous compounds, such as bilirubin, bile acids, and steroid hormones. Additionally, the UGTs glucuronidate exogenous compounds such as opioid analgesics, non-steroidal anti-inflammatory agents (NSAIDs), anticonvulsants, and antiviral drugs [3]. 

Glucuronidation mainly terminates and enhances the elimination of chemical compounds by enhancing their solubility in urine. Additionally, glucuronidated compounds are large, which favors their elimination through biliary excretion [4]. Therefore, the glucuronidation reaction can increase the efficacy and toxicity of some drugs, and glucuronide morphine is reportedly 100 times more potent than the morphine substrate itself [5]. 

Glucuronidation occurs in mammalian species, although significant inter-species differences exist in the rate of glucuronidation, expression, and selectivity [6]. For example, codeine is glucuronidated at higher rates among humans than rats [7,8]. Additionally, cat livers cannot glucuronidate the analgesic paracetamol drug [9]. Therefore, any information obtained about glucuronidation in animals is not directly applicable to humans.

## 2. UGT Isoforms and Genes

The UGT superfamily includes many isoforms with different substrate selectivity and expression [10]. Twenty-two UGTs have been identified in humans [10,11]. Almost all UGT isoforms consist of 29 conserved amino acids involved in the binding to the UDP-glucuronic acid [8]. The UGT isoforms are classified into four major families depending on the DNA sequence similarity: UGT1, 2, 3, and 8 [1]. The UGT1 and 2 families are involved mainly in xenobiotic metabolism, while the UGT3 and 8 families only metabolize endogenous compounds [12]. 

The UGT1 isoform genes consist of five exons. The gene sequence of the first exon is distinct, while they share the remaining four exons. Alternative splicing of the distinct first exons with the common four exons results in the synthesis of nine different isoforms of the UGT1 family; A1 and A3–10 [11]. The isoforms of the UGT2 family contain an entirely different polypeptide sequence; their isoform genes do not share common exons, as in the UGT1 isoforms. The UGT2 family is subdivided into the UGT2A and B subfamilies [13].

## 3. Expression of UGT Isoforms

The liver has the greatest abundance of UGT expression [14,15]. UGTs 1A1, 1A3, 1A4, 1A6, 1A9, 2B7, and 2B15 play major roles in the glucuronidation of drugs in the liver. Additionally, the UGT1A and 2B subfamilies are also expressed in the kidneys, small intestine, colon, stomach, lungs, epithelium, ovaries, testes, mammary glands, prostate, and heart [16,17]. The UGT3 family is not expressed in the liver; it is mainly expressed in the thymus, testes, and kidneys [12]. Therefore, the UGT3 family members are considered extrahepatic UGT enzymes. The UGT2B subfamily isoforms are expressed at higher rates than the UGT1A subfamily isoforms [14,15,17]. UGTs are transmembrane proteins located in the smooth endoplasmic reticulum of cells [18]. 

Many transcriptional factors can regulate the expression of UGT genes. Hepatocyte nuclear factors (HNFs) 1 and 4, the aryl hydrocarbon receptor (AhR), constitutive androstane receptor (CAR), pregnane X receptor (PXR), farnesoid X receptor (FXR), liver X receptor (LXR), and peroxisome proliferator-activated receptors (PPARs) regulate the expression of UGTs in the liver and other tissues [3,19,20]. CAR induces *UGT1A1* and PXR regulates the expression of the *UGT1A1*, *1A3*, *1A4*, and *1A6* genes [21,22]. Activation of FXR upregulates *UGT2B4* and downregulates *UGT2B7* [23,24] and LXR induces the expression of the *UGT1A3* gene [25]. PPARα regulates the expression of the *UGT1A1*, *1A3*, *1A4*, *1A6*, *1A9*, and *2B4* genes in a tissue-specific manner [26]. Furthermore, the *UGT1A1*, *1A3*, *1A4*, *1A6*, and *1A9* genes are upregulated after the activation of AhR nuclear receptor ligands, such as polycyclic aromatic hydrocarbons [27]. Steroid hormones are regulators of UGT expression in the breast and prostate, and 19β-estradiol and dihydrotestosterone increase the expression of UGT genes responsible for glucuronidation of androgens [28]. Furthermore, Jarrar et al. (2019) showed that NSAIDs downregulated the mRNA expression of the mouse *ugt2b1* gene in the liver and kidneys and upregulated the expression of *ugt2b1* in the heart. However, the underlying mechanisms of how NSAIDs regulate the expression of *ugt2b1* in an organ-specific manner remain to be investigated [6]. 

## 4. The Role of UGTs in Xenobiotic Metabolism

UGT1A1, 1A3, 1A4, 1A6, 1A9, and 2B7 play major roles in drug metabolism in humans [3]. UGT1A1 glucuronidates R-carvedilol [29], etoposide [30], B-estradiol [31], ezetimibe [32], and the active metabolite of irinotecan, SN-38 [33]. UGT1A3 glucuronidates ezetimibe [34] and telmisartan [35]. UGT1A4 glucuronidates amitriptyline [36], lamotrigine [37], midazolam [38], olanzapine [39], and trifluoperazine [40]. UGT1A6 metabolizes deferiprone [41] and paracetamol [42] and UGT1A9 glucuronidates propofol [43], entacapone [44], indomethacin [45], mycophenolic acid [46], and oxazepam [47]. UGT2B7 metabolizes carvedilol [29], codeine [48], diclofenac [45], epirubicin [49], flurbiprofen [45], morphine [50], naloxone [51], and zidovudine [52], while UGT2B15 glucuronidates lorazepam [53] and oxazepam [47].

Glucuronidation of certain drugs, such as cyclooxygenase (COX)-2 selective NSAIDs rofecoxib and celecoxib, requires a hydroxyl group on the drug, which is obtained through a cytochrome P450 (CYP450) oxidative reaction [54,55]. However, glucuronidation of many drugs, such as morphine, can be done without the need for the CYP450 oxidation reaction [50].

UGTs also play a role in the metabolism of phytochemical compounds. For example, glycyrrhetinic acid, which is found in licorice, is glucuronidated through UGT1A1, 1A3, 2B4, and 2B7 [56]. The hepatotoxic alkaloid senecionine is glucuronidated by UGT1A4 [57]. This herbal metabolism by UGTs forms part of the drug–herb interaction and influences the metabolism and hence the efficacy of the drugs.

## 5. Factors Affecting UGT Activity

Table 1 summarizes the factors that affect glucuronidation capacity, such as age, gender, diseases, and genetic variants. Owens et al. [58] found that paracetamol glucuronidation was affected by age and renal function. In another study, activity and expression of UGT1A4, the main UGT in paracetamol glucuronidation, differed widely according to age, and the maximum UGT1A4 protein levels peaked at around 20 months old [59]. The expression of UGTs in prenatal children and infants is low, possibly contributing to the susceptibility of neonates to certain drug toxicities [60].

In terms of the effect of human diseases on drug glucuronidation, microsomes isolated from cirrhotic human livers showed reduced glucuronidation capacities for zidovudine and lidocaine [61]. Additionally, glucuronidation was decreased by two-fold in hepatic cancer tissues treated with the anti-hepatic cancer drug sorafenib compared to normal liver tissues, and this was associated with the decreased hepatic protein expression of UGTs [62]. Mouse *ugt2b1* and *ugt1a1* genes were downregulated in the liver of uncontrolled diabetic mice, but this downregulation was normalized after insulin treatment [63]. This may explain, at least in part, the reduced capacity of drug glucuronidation in diabetic patients [64].

Gender affected the drug glucuronidation of (S)-oxazepam [60,65], which was higher in males because males had higher levels of UGT2B15 activity than females. 

**Figure 1:**
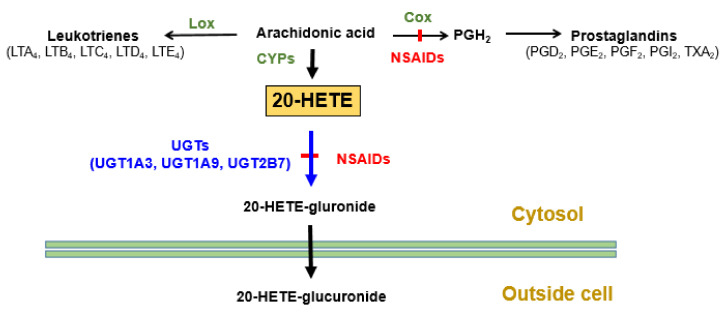
Summary of enzymes affecting 20-HETE synthesis and removal. LTA_4_, Leukotriene A4; LOX, Lipoxygenase; COX, Cyclooxygenase; NSAIDs, Non-steroidal anti-inflammatory drugs; PGH_2_, Prostaglandin H_2;_ TXA_2_, Thromboxane A2; HETE, Hydroxyeicosatetraenoic acid; CYP, Cytochrome P450; UGT, Uridine 5′-diphospho-glucuronosyltransferase.

Genetic variants in UGT genes play major roles in drugs glucuronidation. Multiple UGT genetic polymorphisms (UGT1A8*3, 1A9*3, and 2B7*2) influenced immunosuppressant mycophenolic acid glucuronidation [66]. In addition, UGT2B7*2 reportedly affected tamoxifen plasma levels [67] and was associated with diclofenac-induced hepatic toxicity [68]. Many studies showed that UGT1A1*28 significantly affected the pharmacokinetics and activities of the anticancer drug irinotecan [69,70]. Additionally, using in vitro methods, we showed that the UGT2B7*2 genetic polymorphism reduced 20-hydroxyeicosatetraenoic acid (20-HETE) glucuronidation, and this reduction was increased after incubating liver microsomes with diclofenac, which is a potent NSAID inhibitor of 20-HETE glucuronidation [71]. These results may explain one of the mechanisms underlying NSAID-induced cardiotoxicity. Figure 1 shows the roles of UGTs in the metabolism of the arachidonic acid metabolite 20-HETE.

Enzyme inducers and smoking can alter the glucuronidation of drugs. Rifampin, the PXR nuclear receptor agonist, decreased the plasma levels of the human immune-deficiency virus (HIV) antiviral zidovudine and accelerated its inactivation by glucuronidation [72]. The plasma levels of SN-38, the active metabolite of the anticancer drug irinotecan, were decreased by approximately 40% in smokers [73].

Collectively, many factors affect the glucuronidation of xenobiotic compounds and hence their influences on the human body. Identification of these factors can decrease xenobiotic toxicity and help to optimize drug therapies.

## 6. The Clinical Impact of *UGT1A* Genotype on Drug Response and Toxicity 

*UGT1A1* contains UGT genetic variants with high clinical impacts on drug responses, as illustrated by the Pharmacogenomics Knowledge Base (PharmGKB) website [74]. Patients homozygous for the UGT1A1*28/*28 genotype (rs8175347) and infected with HIV had a higher risk of hyperbilirubinemia after treatment with the protease inhibitor atazanavir [75]. These patients had a TA nucleotide inserted in the promoter region of the *UGT1A1* gene that affects gene expression. The UGT1A1*28 and intronic UGT1A1*6 (rs4148323) alleles increased the likelihood of neutropenia among Asian patients treated with the anticancer drug irinotecan, compared to the wild-type UGT1A1*1 allele [76]. The intronic *UGT1A1 rs34650714* T-allele reduced the metabolism of allopurinol and was associated with a decreased dose of allopurinol in patients with gout [77]. Among cardiovascular patients with angina or heart failure, the UGT1A1*6/*6 genotype had a lower capacity to glucuronidate the beta-blocker carvedilol compared with patients with the wild-type UGT1A1*1/*1 genotype [78].

The *UGT1A3 rs3806596 CC* genotype, with the promotor genetic T > C variant, was associated with hyperbilirubinemia in HIV patients treated with atazanavir and ritonavir [79]. In addition, the intronic *UGT1A3 rs7604115* T-allele was associated with decreased concentrations of plasma montelukast levels in healthy individuals compared to those with the C-allele [80]. Further, the UGT1A3 *2 allele (rs1983023, −751T > C) increased the response to atorvastatin in healthy subjects compared to the wild-type UGT1A3 *1 allele [81]. Additionally, beta-thalassemia patients with the UGT1A3*2 TT genotype had a higher response to deferasirox, as measured by lower liver stiffness values than those with the UGT1A3*2 CC genotype [82].

Colorectal cancer patients with the *UGT1A6 rs2070959 AA* genotype may have an increased risk for severe neutropenia when treated with irinotecan compared to patients with the wild-type *UGT1A6 rs2070959* GG genotype [83]. In addition, pediatric patients with the *UGT1A6 rs6759892 GG* genotype, with a substitution of serine to alanine at amino acid 7 of the UGT1A6 protein sequence, may have an increased likelihood of cardiotoxicity when treated with anticancer anthracyclines compared to patients with the wild-type *UGT1A6* genotype [84]. Additionally, the *UGT 1A6 rs6759892 GG* genotype was associated with adverse drug reactions to deferiprone in patients with beta-thalassemia, as patients with this genotype showed a decreased metabolism of deferiprone [85].

Colorectal cancer patients who carried the non-synonymous UGT1A7*3 T > C rs11692021 allele had an increased risk of vomiting when treated with a combination of anticancer drugs S-1, irinotecan, and oxaliplatin [86]. 

Kidney transplant patients homozygous for the UGT1A8*2 rs1042597 CC genotype had increased diarrhea occurrences when administrated the immune suppressants mycophenolate mofetil and cyclosporine compared to patients with the heterozygous and wild-type genotypes [87]. Patients with epilepsy who had the intronic *UGT1A8 rs2741049 TT* genotype had a lower response to oxcarbazepine than patients with the CC genotype [88].

Non-small cell lung cancer patients with the *UGT1A9 rs3832043 T_9_/T_9_* genotype, which resulted in the deletion of the thymine nucleotide in the −118 promotor sequence of the *UGT1A9* gene, had decreased gene expression and hence reduced glucuronidation capacity of UGT1A9. As a result, these patients showed a reduced elimination rate of the active SN-38 metabolite of irinotecan than those with the wild-type genotype [89]. In addition, hepatotoxicity of a paracetamol overdose was increased in patients with the *UGT1A9 rs8330 CC* genotype [90]. This variant increases the glucuronidation of paracetamol by altering the splicing of exon 5b of UGT1A9 [90]. Table 2 summarizes the major UGT1A genetic variants that have clinical impacts on drug responses.

## 7. The Clinical Impact of the *UGT2B7* Genotype on Drug Responses and Toxicity 

PharmGKB categorizes the *UGT2B7* genetic variants within levels 3 and 4, indicating that further clinical evidence is needed before *UGT2B* genetic variants can be used as biomarkers for drug responses. Patients with the UGT2B7*2 (rs7439366) TT genotype had a reduced response to oxycodone and reduced requirement for codeine compared to the wild-type *UGT2B7* genotype [91]. However, the UGT2B7*2 rs7439366 allele was not associated with increased morphine doses in patients with neoplasms and pain [92]. However, sickle-cell anemia patients with the promotor variant *UGT2B7 rs7668282* TT genotype required a lower morphine dose because they had a higher capacity of morphine glucuronidation compared to patients with the *UGT2B7 rs7668282* CC genotype [93]. Opioid-related disordered patients carrying the *UGT2B7 rs7438135 GG*, *UGT2B7 rs6600880 TT*, and *UGT2B7 rs11940316 TT* genotypes had reduced severity of opiate withdrawal symptoms than patients with the *UGT2B7 rs7438135 AA*, *UGT2B7 rs6600880 AA*, and *UGT2B7 rs11940316 CC* genotypes [94]. Epilepsy patients with the promotor *UGT2B7 rs28365063 −161C > T* genetic variant showed an increased clearance of the antiepileptic drug lamotrigine due to higher gene expression of the *UGT2B7* enzyme [95]. However, epileptic patients with the loss-of-function UGT2B7*2 TT genotype showed an improved oxcarbazepine response due to a reduced oxcarbazepine metabolism rate [96]. The loss-of-function UGT 2B7*3 (rs12233719) G-allele was associated with increased concentrations of valproic acid in the plasma of epilepsy patients compared to patients with the UGT 2B7*3 T-allele [97]. In terms of the influence of the *UGT2B7* genotype on drug-induced toxicity, the intronic *UGT2B7 rs7438135* G-allele was associated with mycophenolate mofetil-induced anemia in kidney transplant patients, whereas the A-allele was not associated with drug-induced toxicity [98]. Table 3 summarizes the reported UGT2B genetic variants and the associated clinical impacts on drug responses.

## 8. The Role of UGTs in Endogenous Metabolism and Susceptibility to Human Diseases

UGTs also play a role in the metabolism of endogenous chemicals, including steroids and unsaturated long-chain fatty acids [99,100]. Besides serving as a substrate for UGTs, these endogenous compounds can also inhibit UGTs. Unsaturated long-chain fatty acids are the most potent inhibitors of several UGT enzymes, including UGT1A3, 1A9, and 2B7 [101]. UGT2B7 metabolizes dietary fatty acids and show inter-individual variations in the glucuronidation of these fatty acids in the intestines [102]. 

Bile acids are common endogenous compounds that undergo glucuronidation. Bile acids are glucuronidated in different human body tissues, but especially in the liver [103]. Biliary glucuronidation is an important pathway in the excretion of bile acids, and impaired biliary secretion leads to hyperbilirubinemia [104]. The UGT2B7*2 genetic variant possibly changes the glucuronidation of chenodeoxycholic acid, affecting the health of individuals [103]. Steroidal hormones are further examples of endogenous compounds that undergo glucuronidation. Sex hormones, thyroxin, and retinoic acid are glucuronidated in different organs [105,106]. Additionally, the UGT1A1*28 genetic variant is associated with plasma estrogen levels in women with breast cancer [107]. Turgeon et al. [108] showed that leukotriene B4 was glucuronidated by UGT1A1, 1A3, 1A4, and 2B7, whereas UGT1A1, 1A3, 1A4, and 1A9 also conjugated most of the HETEs. In addition, the UGT2 family members, especially UGT2B4 and 2B7, conjugated all HETEs. The author suggested that glucuronidation of arachidonic acid metabolites is an irreversible step to inactivate and eliminate endogenous arachidonic acid metabolites from the body. In another in vitro study, arachidonic acid was glucuronidated by UGT1A1, 1A3, 1A4, 1A9, and 1A10, whereas prostaglandin B1 was glucuronidated by UGT1A1, 1A9, and 1A10. All of the arachidonic acid metabolites were glucuronidated by UGT2B7, and arachidonic acid and 20-HETE were the best substrates [109]. UGT1A1, 1A3, 1A9, and 2B7 also glucuronidated 20-HETE [109]. Multiple UGT isoforms are involved in the glucuronidation of arachidonic acid and its metabolites, and these have different enzymatic affinities and maximum capacity rates. As a result, it might be expected that the inhibition or alteration of specific UGT isoforms, such as UGT2B7, have a more significant effect on certain arachidonic acid metabolites, such as 20-HETE and 15-HETE, than other arachidonic acid metabolites. The changes in the arachidonic acid metabolite ratio might affect human homeostasis and lead to a predisposition to certain diseases. Interestingly, one study screened the metabolic and endogenous plasma metabolite changes in human volunteers following administration of diclofenac, a potent UGT2B7 substrate. The results showed that plasma cardiotoxic 20-HETE was significantly increased compared to other endogenous metabolites [110]. In addition, morphine, a strong UGT2B7 inhibitor, altered the arachidonic acid metabolism and activity in a vascular in vitro system [111]. Arachidonic acid metabolites such as 20-HETE were excreted in the glucuronidated form in human urine [112]. Interestingly, the UGT substrate indomethacin reduced urinary 20-HETE levels [113]. It is suggested that inhibition of arachidonic acid-UGT metabolizing enzymes might be one mechanism underlying NSAID-induced hepato- and nephrotoxicity [114]. We showed previously that NSAIDs and the UGT2B7*2 genetic variant inhibited the glucuronidation of 20-HETE [45]. Additionally, NSAIDs inhibited the in vitro glucuronidation of the endogenous hypertensive aldosterone [115] and blood levels of aldosterone increased following NSAID treatment, especially diclofenac and celecoxib [116,117,118]. The increase in aldosterone correlated with decreased aldosterone-glucuronide levels in the urine. Furthermore, in vitro methods showed that diclofenac inhibited testosterone glucuronidation and potentially increased testosterone plasma levels, leading to a hormonal imbalance [119]. The anticonvulsant valproic acid, which causes hormonal imbalance [120], inhibited the endogenous steroidal glucuronidation by inhibiting UGT2B15, the enzyme responsible for steroid metabolism [121]. These data indicate that chemical inhibition of UGTs or loss-of-function genetic variants in the UGT genes can contribute to human disease susceptibility by increasing levels of harmful non-metabolized fatty acids in the plasma, such as 20-HETE. Table 4 summarizes the possible mechanisms of drug-induced toxicity involved in the inhibition of endogenous glucuronidation.

## 9. Conclusions

Chemical inhibition and genetic variants of the UGT genes play important roles in the drug response, toxicity, and susceptibility to human diseases. However, clinical evidence has shown that the UGT1A1 isoform genetic variants can be considered biomarkers for drug responses and susceptibility to diseases. Additionally, inhibition of endogenous glucuronidation can lead to an imbalance in the levels of endogenous fatty acids and steroidal hormones and cause human diseases. Further clinical studies are needed to validate the clinical impacts of the UGT1A and UGT2B genes for personalized medicine and human diseases.

## Figures and Tables

**Table 1:** Factors affecting glucuronidation capacity.

Factor	Effect on Glucuronidation	References
Age	Neonates have a low capacity for drug glucuronidation, such as paracetamol, due to low expression of UGT enzymes. The expression and activity of UGTs reach maximum at around 20 months of age.	[58,59,60]
Disease	Liver cirrhosis, cancer, and diabetes mellitus decrease glucuronidation capacity.	[61,62,63]
Gender	Males have higher glucuronidation activity against (S)-oxazepam than females.	[64,65]
Genetic variants	UGT2B7*2 decreases glucuronidation capacity towards mycophenolic acid and fatty acids, such as arachidonic acid metabolites. UGT1A1*28 decreases the metabolism of irinotecan.	[66,67,69,70]
Environmental	Smoking induces the UGT1A family, which increases the metabolism of SN-28.	[73]

**Table 2:** UGT1A genetic variants with reported clinical impacts on drugs responses.

Genetic Variant	Rs Number	Clinical Impact on Drug Responses	References
*UGT1A1 (TA)_6_TAA> (TA)_7_TAA* (UGT1A1 *28)	rs8175347	Associated with increased hyperbilirubinemia after treatment with the protease inhibitor atazanavir.	[75]
*UGT1A1 211G > A* (UGT1A1*6)	rs4148323	The UGT1A1*6 allele increases the likelihood of neutropenia among Asian patients treated with the anticancer drug irinotecan. In addition, UGT1A1*6 can affect the metabolism of carvedilol.	[76,78]
*UGT1A3 −66T > C*	rs3806596	Associated with hyperbilirubinemia in HIV patients treated with atazanavir and ritonavir.	[79]
*UGT1A3 IVS1 −17564C > T*	rs7604115	The *UGT1A3T* allele is associated with decreased concentrations of plasma montelukast levels in healthy individuals.	[80]
*UGT1A3 −751T > C* (UGT1A3 *2)	rs1983023	The UGT1A3*2C allele increases the response to atorvastatin in healthy subjects compared to the wild-type UGT1A3 *1 allele. The UGT1A3*2T allele can increase the response to deferasirox.	[81,82]
*UGT1A6 A > G* (UGT1A6*5)	rs2070959	Can increase the risk for severe neutropenia among patients on irinotecan treatment.	[83]
*UGT1A6 19A > G*	rs6759892	May increase the risk of cardiotoxicity of anticancer anthracyclines. In addition, this genetic variant is associated with adverse drug reactions to deferiprone in patients with beta-thalassemia.	[84,85]
*UGT1A7622T > C* (UGT1A7*3)	rs11692021	UGT1A7*3 may increase the risk of vomiting when treated with a combination of anticancer drugs S-1, irinotecan, and oxaliplatin.	[86]
*UGT1A8 518C > G* (UGT1A8*2)	rs1042597	This genetic variant can increase the risk of diarrhea among patients with kidney transplants on immune suppressant treatment.	[87]
*UGT1A8 I399C > T*	rs2741049	Can lower the response to oxcarbazepine among epileptic patients.	[88]
*UGT1A9-118T_10_/T_9_*	rs3832043	The *UGT1A9T_9_* variant decreased the elimination rate of the active metabolite of irinotecan SN-38 in non-small cell lung cancer patients.	[89]

**Table 3:** UGT2B genetic variants with reported clinical impacts on drug responses.

Genetic Variant	Rs Number	Clinical Impacts on Drug Responses	References
*UGT2B7802C > T* (UGT2B7*2)	rs7439366	Can decrease the response to oxycodone and the dosage of codeine. Additionally, can decrease oxcarbazepine metabolism.	[91,96]
*UGT2B7 −840C > T*	*rs7668282*	The *UGT2B7 rs7668282* TT genotype is associated with decreased morphine glucuronidation capacity.	[93]
*UGT2B7 −900G > A*	rs7438135	Patients with the *UGT2B7 rs7438135 G*-allele have a reduced severity of opiate withdrawal symptoms than those with the wild-type A-allele. Additionally, the *UGT2B7 rs7438135 G*-allele was associated with mycophenolate mofetil-induced anemia in kidney transplant patients.	[94,98]
*UGT2B7 −1759A > T*	rs6600880	Patients with the *UGT2B7 rs6600880 A*-allele may have a reduced severity of opiate withdrawal symptoms than those with the wild-type A-allele.	[94]
*UGT2B7 −1112C > T*	rs11940316	Patients with the *UGT2B7 rs11940316 T*-allele may have a reduced severity of opiate withdrawal symptoms than those with the wild-type C-allele.	[94]
*UGT2B7 −161C > T*	*rs28365063*	The *UGT2B7 rs28365063 T*-allele is associated with increased clearance of the antiepileptic drug lamotrigine.	[95]
*UGT 2B7 211G > T* (UGT 2B7*3)	rs12233719	The UGT 2B7*3 G-allele is associated with increased valproic acid concentrations in the plasma.	[97]

**Table 4:** Mechanisms of drug-induced toxicity involved in the inhibition of endogenous glucuronidation.

Drugs	Potential Toxicity	Mechanisms	References
NSAIDs	Elevation of blood aldosterone levels that increase water reabsorption.	Inhibition of aldosterone glucuronidation by inhibiting UGT2B7 and 15.	[116,117,118]
NSAIDs	Elevation of blood cardiotoxic 20-HETE levels.	Inhibition of 20-HETE glucuronidation by inhibiting UGT2B7, 1A3, and 1A9 isoforms.	[71]
Valproic acid	Imbalance of blood steroidal hormones.	Inhibition of UGT2B15.	[121]
Diclofenac	Elevation of testosterone levels.	Inhibition of testosterone glucuronidation.	[119]

## Data Availability

Not applicable.
